# Systemic Lupus Erythematosus

**DOI:** 10.1155/2012/437282

**Published:** 2012-09-20

**Authors:** Chaim Putterman, Roberto Caricchio, Anne Davidson, Harris Perlman

**Affiliations:** ^1^Division of Rheumatology, Montefiore Medical Center, Bronx, NY 10467, USA; ^2^Division of Rheumatology, Albert Einstein College of Medicine, Bronx, NY 10461, USA; ^3^Section of Rheumatology, Temple University School of Medicine, Philadelphia, PA 19140, USA; ^4^Center for Autoimmune and Musculoskeletal Diseases, The Feinstein Institute for Medical Research, Manhasset, NY 11030, USA; ^5^Division of Rheumatology, Department of Medicine, Feinberg School of Medicine, Northwestern University, Chicago, IL 60611, USA

## 1. Introduction

Systemic lupus erythematosus is a systemic autoimmune disease with a worldwide distribution. Although both men and women of all age groups can be affected, women outnumber men almost 10 fold and the typical lupus patient is a young woman during her reproductive years. Clinically, lupus is a disease with an unpredictable course involving flares and remissions, where cumulative damage over time significantly interferes with the quality of life and adversely affects organ function. Multiple cells, tissues, and organs can be affected in this disease, and the clinical picture can vary greatly between patients. Indeed, even in the same patient the clinical picture may not be consistent over time. Organ systems most commonly involved in lupus patients include joints, skin and mucous membranes, blood cells, brain, and kidney.

Although the prognosis of lupus patients has dramatically improved with the widespread introduction of potent immunosuppressive therapies and better medical management of acute disease exacerbations, a diagnosis of SLE remains associated with an appreciably shortened life span. Moreover, the mortality rates are still significant among patients with active disease. With more lupus patients living with chronic, intermittently active disease, it has become evident that there is significantly accelerated atherosclerotic cardiovascular disease that is insufficiently explained by traditional risk factors.

A second major cause of mortality in SLE is infection. Lupus patients have an inherent susceptibility to infections due to their disease. In addition, the major side effect of the large majority of medications currently used for treatment of lupus is immunosuppression, which confers a greatly increased risk for infections with typical and atypical organisms. For this reason, the use of more aggressive approaches is usually restricted to patients with active disease, with lower doses of immunosuppressive treatment being used for chronic maintenance.

The universal belief and expectation among investigators and physicians involved in SLE is that a more comprehensive and accurate understanding of the underlying mechanisms of disease will lead to the development of more targeted therapies. Such novel approaches to treatment would presumably result in improved patient response rates, decreased numbers of flares, attenuated cumulative damage, and enhanced preservation of organ function over time. Moreover, even if newer therapies have a similar efficacy profile to medications in current use, the employment of more targeted and specific therapeutic modalities could reasonably result in less unintended side effects. In this special issue, we have gathered contributions from physicians and researchers from North America, South America, Europe, and Asia that highlight several important and/or novel aspects of the molecular pathogenesis, clinical organ involvement, and experimental therapies in this prototypical systemic autoimmune disease.

## 2. Disease Pathogenesis

Gender and hormones play a crucial role in SLE: the disease is much more common in females, and its presentation often correlates with changes in estrogen and/or progesterone levels. Nonhormonal, X-chromosome-related contributions may be important as well. The contribution of gender to the prevalence of disease, types of clinical manifestations, and pathogenesis are summarized in this issue by J. Schwartzman et al. The genetics of SLE are being unraveled by the use of genomewide association studies (GWAS) that have uncovered the role of multiple genetic polymorphisms, each of which confers a modest increase (<2 fold) in an individual's risk for lupus. Nevertheless, in all these studies an association with particular MHC alleles remains the major genetic contributor, conferring a 3-4 fold risk for lupus. M. Relle and A. Schwarting review the role of MHC-linked susceptibility genes in experimental and human disease. A. H. Draborg et al. remind us of the importance of infection in triggering lupus autoimmunity in the genetically susceptible host, particularly the Epstein-Barr virus, which has been epidemiologically linked to SLE onset.

The immune pathogenesis of SLE is complex and remains a matter of considerable study and debate. Although initially lupus was believed to be a disease of the adaptive immune response, a growing recognition of the crosstalk between the innate and adaptive arms of the immune system in recent years, the discovery of several families of pattern recognition receptors, and the suggestive role of type I IFN pathway uncovered by GWAS studies, have led to an increasing realization that innate immune cells and effectors are central contributors to the pathogenesis of SLE. Moreover, there is abundant evidence from animal models that experimental lupus can be induced by individual aberrations in a multitude of cell types and cellular pathways, suggesting that the pathogenesis of lupus probably varies between individuals. Furthermore, the operative mechanisms may be different during the triggering, amplification, persistence, and flare phases of the disease.

Lupus research has benefited tremendously from the rapid pace of advances in the field of immunology at large. Considering the intricate and multifaceted pathogenesis of SLE, it is not surprising that advances in basic immunology are often followed by studies investigating the relevance of this particular mechanism in the pathogenesis of lupus. In this issue, the role of some of these new players in SLE is critically examined. Y.-P. Chuang et al. investigate invariant NK T cells and their modulatory function, while A. Alunno et al. provide new insights into the balance between T_H_17 and regulatory T cells. Finally, A. P. Alegretti et al. demonstrate decreased complement regulatory proteins on lupus peripheral blood mononuclear cells, a finding which may be relevant to pathogenesis and may also serve as a disease biomarker.

## 3. Organ Damage

Kidney disease is a particularly important manifestation of lupus. Renal manifestations appear in a high percentage of patients, and even if treated with aggressive therapy can lead to progressive renal failure and end-stage renal disease. Indeed, renal involvement and its associated complications stubbornly remain one of the major causes of morbidity and mortality in lupus patients. Although T cells, macrophages, cytokines, and chemokines, among many other immune mediators, are important in nephritis initiation and/or progression, B cells and autoantibodies are known to play an instrumental role in the pathogenesis of lupus nephritis. S. Yung and T. M. Chan review several of the main types of lupus autoantibodies closely associated with nephritis and the results of the interactions of these pathogenic antibodies with resident kidney cells, while Y. Shen et al. demonstrate that the presence of intrarenal B cell infiltrates may be a significant prognostic factor in human lupus nephritis.

Two other specific (albeit less common) types of disease manifestations which are receiving increasing attention, namely, pulmonary hypertension and gastrointestinal involvement, are also addressed in this issue by A. Dahla and Y. Yang et al., respectively.

## 4. Therapies

There has been much recent interest in therapies that enhance immune regulation as a means of normalizing tolerance defects in autoimmunity. Autologous mesenchymal stem cell transplantation is engendering interest as a potential treatment for several types of immunologically mediated disease. Its potential role as a treatment for lupus is reviewed in this issue by D. Wang et al. and A. Darmont. One caveat of this approach is that lupus mesenchymal stem cells have signaling abnormalities, as demonstrated by Y. Tang et al.

Murine lupus models have many similarities to human disease, and serve as valuable in vivo laboratories for proof-of-concept therapeutic studies. One such new approach is tested by Y. W. Jiang et al. in the lupus prone MRL/lpr mouse strain.

## 5. Summary

Lupus remains a puzzling disease with protean manifestations that has so far been disappointingly resistant to new forays into biologic therapies based on rational immune approaches. Clearly, much headway has been made in our understanding of pathogenic mechanisms and many promising targeted approaches are being tested both in animal models of disease and in human trials. Nevertheless, the heterogeneity of disease mechanisms, clinical manifestations, and pathologic findings makes the design of clinical trials particularly challenging. The contributors to this issue have identified a number of expanding research areas that continue to yield new insights into pathogenesis and treatment of lupus. An increase in this knowledge will be required to develop therapies that can prevent and treat disease without the excessive toxicities of our current armamentarium.

*Chaim Putterman* *Roberto Caricchio* *Anne Davidson* *Harris Perlman*

